# Chronic Obstructive Pulmonary Disease-Derived Circulating Cells Release IL-18 and IL-33 under Ultrafine Particulate Matter Exposure in a Caspase-1/8-Independent Manner

**DOI:** 10.3389/fimmu.2017.01415

**Published:** 2017-10-26

**Authors:** Gianluigi De Falco, Chiara Colarusso, Michela Terlizzi, Ada Popolo, Michela Pecoraro, Mario Commodo, Patrizia Minutolo, Mariano Sirignano, Andrea D’Anna, Rita P. Aquino, Aldo Pinto, Antonio Molino, Rosalinda Sorrentino

**Affiliations:** ^1^Dipartimento di Ingegneria Chimica, dei Materiali e della Produzione Industriale, Università degli Studi di Napoli Federico II, Naples, Italy; ^2^Department of Pharmacy, University of Salerno, Fisciano, Italy; ^3^ImmunePharma s.r.l., University of Salerno, Fisciano, Italy; ^4^Drug Discovery and Development Program, Department of Pharmacy, University of Salerno, Fisciano, Italy; ^5^Institute for Research on Combustion (CNR), Naples, Italy; ^6^Department of Respiratory Medicine, Respiratory Division, University of Naples Federico II, Naples, Italy

**Keywords:** combustion-generated ultrafine particles, inflammation, airway disease, chronic obstructive pulmonary disease, peripheral blood mononuclear cells

## Abstract

Chronic obstructive pulmonary disease (COPD) is considered the fourth-leading causes of death worldwide; COPD is caused by inhalation of noxious indoor and outdoor particles, especially cigarette smoke that represents the first risk factor for this respiratory disorder. To mimic the effects of particulate matter on COPD, we isolated peripheral blood mononuclear cells (PBMCs) and treated them with combustion-generated ultrafine particles (UFPs) obtained from two different fuel mixtures, namely, pure ethylene and a mixture of ethylene and dimethylfuran (the latter mimicking the combustion of biofuels). UFPs were separated in two fractions: (1) sub-10 nm particles, named nano organic carbon (NOC) particles and (2) primarily soot particles of 20–40 nm and their agglomerates (200 nm). We found that both NOC and soot UFPs induced the release of IL-18 and IL-33 from unstable/exacerbated COPD-derived PBMCs. This effect was associated with higher levels of mitochondrial dysfunction and derived reactive oxygen species, which were higher in PBMCs from unstable COPD patients after combustion-generated UFP exposure. Moreover, lower mRNA expression of the repairing enzyme OGG1 was associated with the higher levels of 8-OH-dG compared with non-smoker and smokers. It was interesting that IL-18 and IL-33 release from PBMCs of unstable COPD patients was not NOD-like receptor 3/caspase-1 or caspase-8-dependent, but rather correlated to caspase-4 release. This effect was not evident in stable COPD-derived PBMCs. Our data suggest that combustion-generated UFPs induce the release of caspase-4-dependent inflammasome from PBMCs of COPD patients compared with healthy subjects, shedding new light into the biology of this key complex in COPD.

## Introduction

Chronic obstructive pulmonary disease (COPD) is considered the fourth-leading causes of death worldwide, predicted to become the fifth ranked cause of disability worldwide (1). The development of COPD is caused by inhalation of noxious indoor and outdoor particles (2), especially cigarette smoke that represents the first risk factor for this respiratory disorder (3). Herein, chronic inflammatory pattern/s observed in the lung of COPD patients derives from the exposure of both cigarette smoke and air pollution, which affect lung resident and circulating cells (1, 3, 4). Although, the molecular mechanisms causing inflammatory patterns in COPD patients are still elusive; so far, an altered immune response followed by chronic inflammation has been described at the basis of COPD (1–3, 5, 6). Therefore, the main goal of our study was to understand the pro-inflammatory pattern induced by the exposure to ultrafine particles (UFPs) of human peripheral blood mononuclear cells (PBMCs) obtained from COPD patients, who are all former or current smokers. Over recent years, several papers demonstrated that oxidative stress due to cigarette smoking is critical for the activation of molecular mechanisms, such as NF-kB activation, that can lead to the release of pro-inflammatory mediators (3). In the milieu of pro-inflammatory mediators, IL-1-like cytokines have been detected in both sputum and bronchoalveolar lavage fluid of COPD patients (1) and of experimental murine models (7–9). It has been observed that biomass fuel smoke induces the activation of the inflammasome, a multimeric complex, that leads to the release of IL-1-like cytokines, which are responsible of early inflammation and that can result in a long-term chronic inflammation (10). Emerging evidence suggests that NOD-like receptor 3 (NLRP3) inflammasome may be involved in COPD pathogenesis (1). We previously demonstrated that ultrafine carbonaceous particulate matter (UFP) generated in very fuel-rich combustion conditions (mean sizes of 100 nm) triggered NLRP3/caspase-1-dependent inflammasome in smoker-derived PBMCs (11). To date, high levels of IL-1β are found in the lungs of patients with COPD after cigarette smoke exposure, implying the involvement of the inflammasome in this pathology. In support, several cigarette exposure COPD animal models showed that the genetic absence of both caspase-1, the enzyme involved in the canonical inflammasome complex, and NLRP3 reduced COPD-like features in mice (8, 9, 12), and this observation was further confirmed by neutralization of IL-1β (13). However, published experimental data on human samples from COPD patients are controversial in this regard (1). While several clinical studies have shown that IL-1-like cytokine levels are elevated in the lungs of patients with COPD (1), others found no correlation between NLRP3, caspase-1, and IL-1β responses when comparing stable COPD patients to smokers (14). Nevertheless, the latter study was performed on a cohort of stable COPD patients compared with healthy smokers. Di Stefano et al. proposed that, rather, the inflammasome complex could be relevant during COPD exacerbations (15).

In this article, we analyzed the effect of UFPs representative of those encountered in practical combustion conditions, including gasoline and diesel engines, and we found that PBMCs obtained from unstable/exacerbated COPD patients were able to solely release IL-18 and IL-33, but not IL-1α and IL-1β, when treated with combustion-formed UFPs. By contrast, PBMCs obtained from smoker and non-smoker healthy volunteers were less susceptible than unstable COPD-derived PBMCs to biofuel-like UFP exposure. Moreover, this effect was not dependent on NLRP3, caspase-1, and caspase-8, but rather on the release of caspase-4, implying the involvement of the non-canonical inflammasome pathway in PBMCs obtained from unstable/exacerbated COPD patients. By contrast, PBMCs obtained from stable COPD patients were not affected by the exposure to this particulate matter in terms of IL-18 and IL-33 release.

## Materials and Methods

### Human Samples

Blood from COPD and non-COPD subjects were collected at the Hospital “Monaldi-Azienda Ospedaliera (AORN)-Ospedale dei Colli” in Naples, Italy, after signed informed consent. The experimental protocol was performed in accordance with the guidelines and regulations provided and accepted by the Ethical Committee of the “Monaldi-AORN-Ospedale dei Colli” Hospital (approval number 1254/2014). COPD and non-COPD subjects were 60 ± 10 (mean ± SEM) years of age. COPD subjects were smokers or former smokers; non-COPD subjects were divided in smokers and non-smokers. Blood was collected and used within 24 h.

### Isolation of Human PBMCs

Mononuclear cells were isolated according to Ficoll’s protocol as already reported (11). Briefly, blood (5 ml) was mixed with cell medium (5 ml) supplemented with sole antibiotics and Ficoll medium (Life Sciences, Italy). PBMC layer was collected, and platelets were separated by centrifugation at 150 *g* for 10 min. PBMCs were then collected in cell medium, plated (10^5^ cells/well) and treated for 1, 3, or 5 h, accordingly. Treatment was performed in duplicate for each patient. Experimental time points were repeated for each patient.

### Preparation of UFPs

Ultrafine particles were collected from laboratory premixed flames, which were run in fuel-rich conditions feeding ethylene/air and ethylene-2,5-dimethylfuran/air mixtures with an equivalence ratio of 2.0 and divided in two parts according to their sizes: sub-10 nm particles and larger soot particles (16).

Sub-10 nm particles were mostly constituted of organic carbon (17). They were stacks of few aromatic molecules, which had high-molecular mass and were constituted by four to six fused benzene rings with a dimension of about 1.2 nm connected by chain-like bridge (van der Waals interactions).

Larger soot particles were more graphitic carbon structures (17); they had sizes ranging from 20–40 nm, typical of the primary soot particles, to 100–200 nm typical of the chain-like aggregates of the primary particles: ultraviolet–visible and infrared spectroscopy and Raman spectroscopy were used for structural analysis of the carbon-network constituting the particles (18, 19). Soot particles appeared as a network of aromatic structures with few peripheral H atoms. Elemental analysis confirmed the low presence of H atoms in the soot particles, as the amount of C was approximately 95–98%, in mass, of the total material, and H was approximately 1–2%, with the rest being trace compounds, possibly oxygen.

Sub-10 nm particles and primary soot particles and aggregates were dispersed in bidistilled water to obtain a suspension with a concentration of 5 ppm (5 µg/ml). The mass concentrations were determined assuming a density of 1.2 and 1.8 g/cm^3^ for sub-10 nm and soot particles, respectively. Here, we define NOC-E and NOC-ED the samples of sub-10 nm particles collected burning ethylene and the ethylene/dimethylfuran, whereas Soot-E and Soot-E/DMF the larger sizes soot particles collected burning ethylene and the ethylene/dimethylfuran, respectively. A summary of the particle characteristics is reported in Table 1.

**Table 1:** Characteristics of the ultrafine particles (UFPs).

UFP characteristics					

Name	Fuel	Particle size	H/C atomic	% O	Aromatic domain dimension
NOC-E	Ethylene	sub-10 nm	0.6–0.8	n.d.	1.0–1.2 nm
NOC-ED	Ethylene/2,5 DMF (80/20 vol.%)	sub-10 nm	0.6–0.8	n.d.	1.0–1.2 nm
Soot-E	Ethylene	20–200 nm	0.2–0.3	<1%	1.2–1.4 nm
Soot-E/DMF	Ethylene/2,5 DMF (80/20 vol.%)	20–200 nm	0.2–0.3	<5%	1.2–1.4 nm

*n.d., not detected; NOC, nano organic carbon; 2,5 DMF, 2,5 dimethylfuran*.

### Cytokine Measurements

IL-18 and IL-33 were measured in cell-free supernatants (75 × 10^4^ cells/well) using commercially available ELISA kits (eBioscience, CA, USA). No differences in cytokine release were observed according to stage of COPD patients. 8-OH-dG was measured following manufacturer’s instructions after 3 h of treatment (5 × 10^6^ cells/well) (Elabscience, USA). The release of caspase-4 was analyzed by an ELISA kit patented by ImmunePharma s.r.l. (RM2014A000080 and PCT/IB2015/051262) (Department of Pharmacy, University of Salerno, Italy). According to the patent policy, and because it is still not commercially available, it is not at the moment possible to describe the technical approach to determine the release of caspase-4 by using the ImmunePharma’s ELISA kit.

### Calcium Measurement

Intracellular Ca^2+^ concentrations ([Ca^2+^]i) were measured as previously reported after 1 h of treatment (5 × 10^3^ cells/well) (20). Data were expressed as percentage of delta increase of fluorescence ratio (F340/F380 nm) induced by ionomycin (1 µM) or carbonyl cyanide p-trifluoromethoxy-phenylhydrazone (FCCP, 0.05 µM)—basal fluorescence/basal fluorescence ratio (F340/F380 nm).

### Flow Cytometry

Peripheral blood mononuclear cells were stained for flow cytometry analysis (BD FACSCalibur Milan, Italy) using the following antibodies: CD14-PE and NLRP3-PeCy5.5. Healthy non-smoker-, smoker-, and COPD-derived PBMCs (2 × 10^5^ cells/well) were stained for MitoSOX Mitochondrial Superoxide Indicator as indicated in the manufacturer’s guide (Life Technologies, USA).

### RT-PCR

Total RNA was isolated from PBMCs (10^7^ cells/well) by using the RNA extraction kit (Qiagen, Milan, Italy). Reverse Transcription was performed by using first-strand cDNA synthesis kit (Qiagen, Milan, Italy) followed by PCR. Thermal cycling conditions were as follow:

1. 5 min at 95°C, followed by 40 cycles of 30 s at 95°C, 60 s at 54°C, 30 s at 60°C for OGG1.
2. 5 min at 95°C, followed by 40 cycles of 30 s at 95°C, 60 s at 58°C, 30 s at 68°C for caspase-4.

Primer pairs were as follow:

OGG1: Forward 5′-GACAAGACCCCATCGAATGC-3′
Reverse 5′-AGCTTCCTGAGATGAGCCTC-3′
CASPASE-4: Forward 5′-TCCCTGGGCAAAGATTTCCT-3′
Reverse 5′-GTCCAGCCTCCATATTCGGA-3′
β-actin: Forward 5′-ACTCTTCCAGCCTTCCTTCC-3′
Reverse 5′-CGTACAGGTCTTTGCGGATG-3′

### Statistical Analysis

Data are reported as median ± interquartile range or as mean ± SEM. Statistical differences were assessed with one-way analysis of variance followed by Bonferroni’s multiple comparison post test, and *p* values less than 0.05 were considered significant.

## Results

### Combustion-Generated UFPs Induced the Release of IL-18 from Human PBMCs Obtained from Unstable COPD Patients

**Figure 1:**
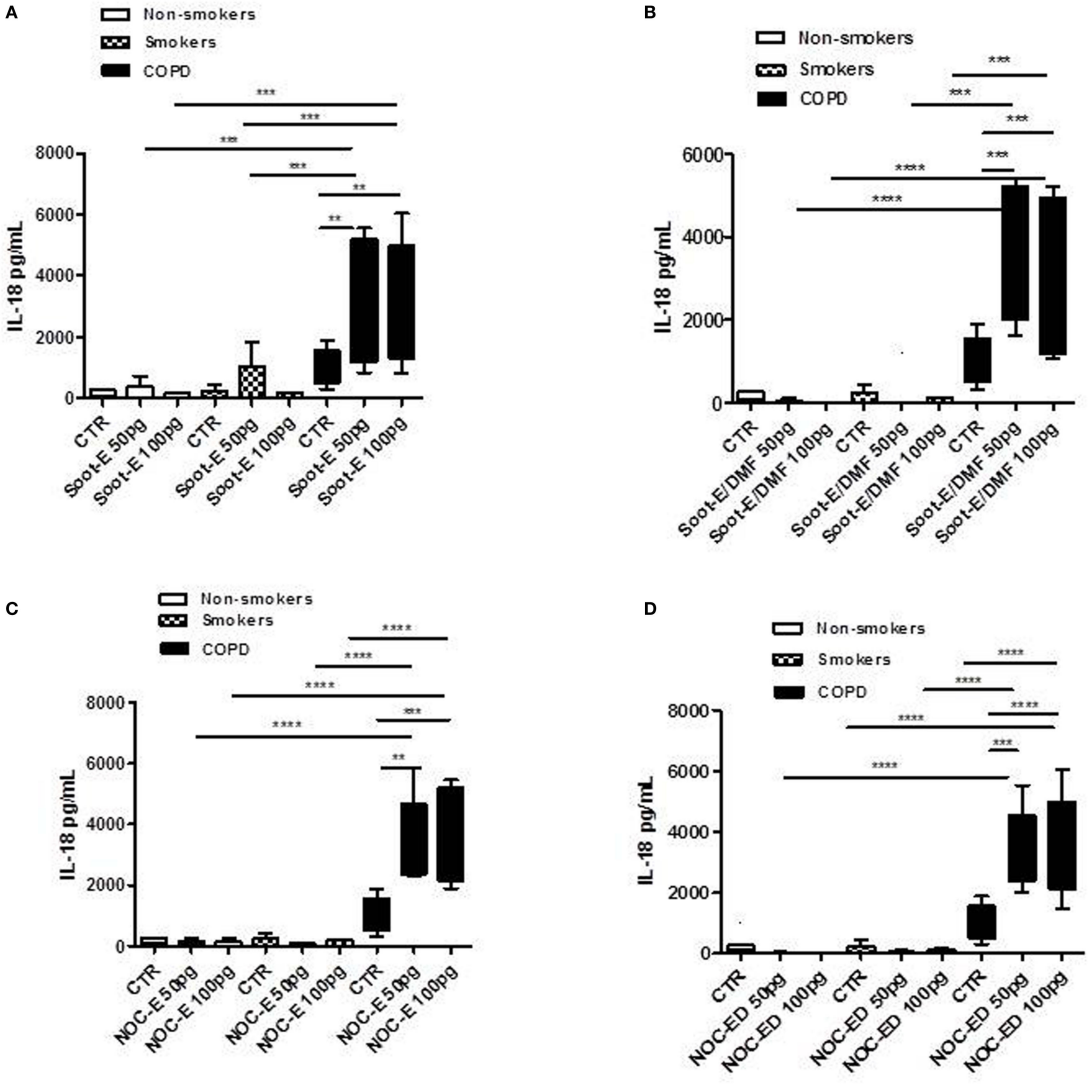
The administration of combustion-generated ultrafine particles (UFPs) for 5 h induced the release of IL-18 by peripheral blood mononuclear cells (PBMCs) obtained from unstable/exacerbated chronic obstructive pulmonary disease (COPD) patients. Healthy non-smoker (white bars), smoker (dotted bars), and COPD (black bars)-derived PBMCs were treated with soot combustion-derived UFPs for 5 h. The addition of Soot-E **(A)**, Soot-E/DMF **(B)**, nano organic carbon (NOC)-E **(C)**, and NOC-ED **(D)** at the concentration of 50–100 pg/ml induced the release of IL-18. Control (CTR) represents untreated cells. Data are presented as median ± interquartile range (*n* = 7). Statistically significant differences were determined by one-way analysis of variance followed by Bonferroni’s multiple comparison posttest. **, ***, and **** represent *p* < 0.01, *p* < 0.0005, and *p* < 0.0001, respectively.

To mimic environmental pollution, PBMCs were isolated from smokers, non-smokers, and COPD patients, and then treated with two classes of combustion-generated UFPs for 5 h. Figure 1 reports the release of IL-18 after the addition of Soot-E (A), Soot-E/DMF (B), NOC-E (C), and NOC-ED (D) at concentrations of 50 and 100 pg/ml. Clearly, UFPs induced the release of IL-18 from PBMCs obtained from unstable exacerbated COPD patients (black bars, Figure 1). To note, the release of IL-18 from PBMCs of unstable/exacerbated COPD patients was significantly higher than that observed from PBMCs of non-smoker (Figure 1, white bars) and smoker (Figure 1, dotted bars) volunteers. Noticeably, Soot-E (Figure 1A) and Soot-E/DMF (Figure 1B) did not induce IL-18 release from PBMCs obtained from non-smoker (Figures 1A,B, white bars) and smokers (Figures 1A,B, dotted bars), compared with the control (CTR)/basal levels.

Similarly, the same mass concentrations of NOC-E (Figures 1C) and NOC-ED (Figure 1D) increased the release of IL-18 from PBMCs obtained from unstable COPD (Figures 1C,D, black bars) and were almost non-active at inducing IL-18 release from non-smoker- derived (white bars) and smoker-derived (dotted bars) PBMCs (Figures 1C,D).

The same mass concentrations of NOC and soot particles correspond to different surface area of the particles exposed to cells due to the different sizes of the particles. In particular, 100 pg/ml of soot have a surface area of 2.2E−4 cm^2^/ml whereas the same amount of NOC has a surface area of 2E−3 cm^2^/ml, implying that NOC particles expose higher surface area than soot particles, due to their lower size. To verify, whether the size of these sub-10 nm particles was a limitation, we treated cells with coronene and pyrene, polycyclic aromatic hydrocarbons with sizes comparable to those measured for the aromatic compounds that constitute the nanoparticles (1 pg/ml up to 1 ng/ml). As shown in Figures S1A–C in Supplementary Material, both coronene and pyrene (10 pg/ml) significantly increased IL-18 release from PBMCs of non-smokers (Figure S1A in Supplementary Material), smokers (Figure S1B in Supplementary Material), and unstable COPD (Figure S1C in Supplementary Material) at 10 pg/ml corresponding to 6E−4 cm^2^, implying that the size of the particles was not as relevant as the nature of the particles. Moreover, similarly to NOC-E and NOC-ED particles, we observed that cells were more responsive to the lower concentrations than the higher (data not shown), reaching a plateau at 10 pg/ml. Interestingly, PBMCs from non-smokers (Figure S1A in Supplementary Material) and smokers (Figure S1B in Supplementary Material) were less responsive in terms of IL-18 release compared with PBMCs obtained from unstable COPD patients (Figure S1C in Supplementary Material), which instead showed a significant increase. These data underlie that the organic nature of UFPs, as well as soot and NOC, is of relevant importance for cell responsiveness in terms of IL-18 release.

### Combustion-Generated UFPs Induced the Release of IL-33 from Human PBMCs Obtained from Unstable/Exacerbated COPD Patients

**Figure 2:**
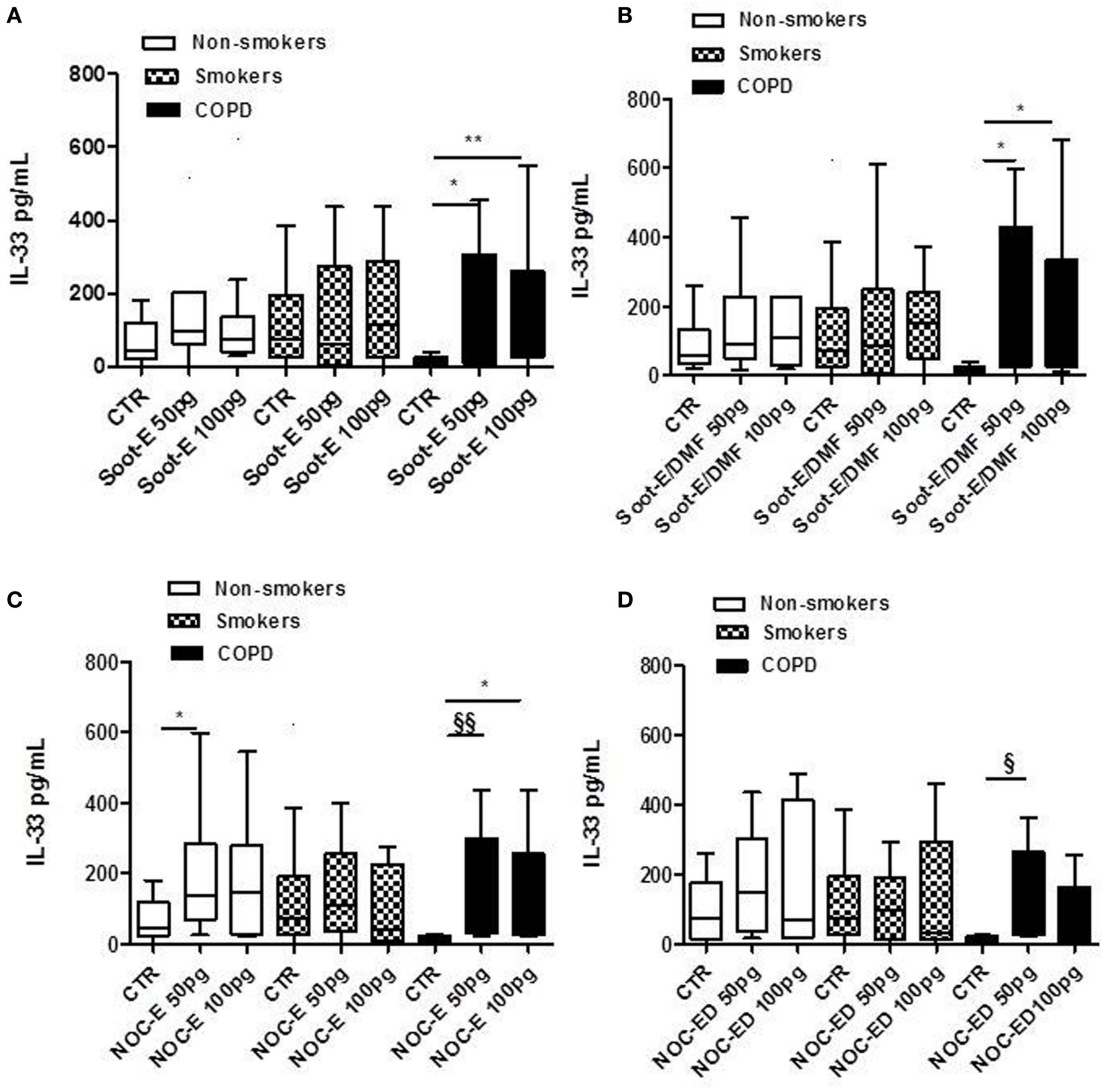
The administration of combustion-generated ultrafine particles (UFPs) for 5 h induced the release of IL-33 by peripheral blood mononuclear cells (PBMCs) obtained from unstable/exacerbated chronic obstructive pulmonary disease (COPD) patients. Healthy non-smoker (white bars), smoker (dotted bars), and COPD (black bars)-derived PBMCs were treated with soot combustion-derived UFPs for 5 h. The addition of Soot-E **(A)**, Soot-E/DMF **(B)**, NOC-E **(C)**, and NOC-ED **(D)** at the concentration of 50–100 pg/ml induced the release of IL-33. Control (CTR) represents untreated cells. Data are presented as median ± interquartile range M (*n* = 7). Statistically significant differences were determined by one-way analysis of variance followed by Bonferroni’s multiple comparison posttest. *, **, ^§^, and ^§§^ represent *p* < 0.05, *p* < 0.01, *p* < 0.005, and *p* < 0.001, respectively.

Similarly to what performed for IL-18, we treated PBMCs from the three cohorts with soot and NOC UFPs. The addition of Soot-E (Figure 2A), Soot-E/DMF (Figure 2B, black bars), NOC-E (Figure 2C, black bars), and NOC-ED (Figure 2D, black bars) significantly increased the release of IL-33 at the concentration of 50–100 pg/ml from PBMCs obtained from unstable/exacerbated COPD patients. Also in this case, we observed that IL-33 release reached a plateau at 50–100 pg/ml whereas at higher concentrations of these UFPs (>100 pg/ml up to 1 ng/ml, data not shown) no increasing effect was observed. However, it is to note that despite what happened for IL-18, no statistical increase of IL-33 release was observed comparing results among the groups represented by non-smokers (Figure 2, white bars) and smokers (Figure 2, dotted bars). Instead, the stimulation of PBMCs derived by unstable COPD patients with Soot-E (Figure 2A, black bars), Soot-ED (Figure 2B, black bars), NOC-E (Figure 2C, black bars), and NOC-ED (Figure 2D, black bars) increased the levels of IL-33. It is to note, though, that we observed a statistical difference in IL-33 basal levels that were higher in smokers than unstable COPD-derived PBMCs (Figure 2, dotted bars vs black bars).

### Combustion-Generated UFPs Increased Oxidative Stress in Human PBMCs Obtained from Unstable/Exacerbated COPD Patients

**Figure 3:**
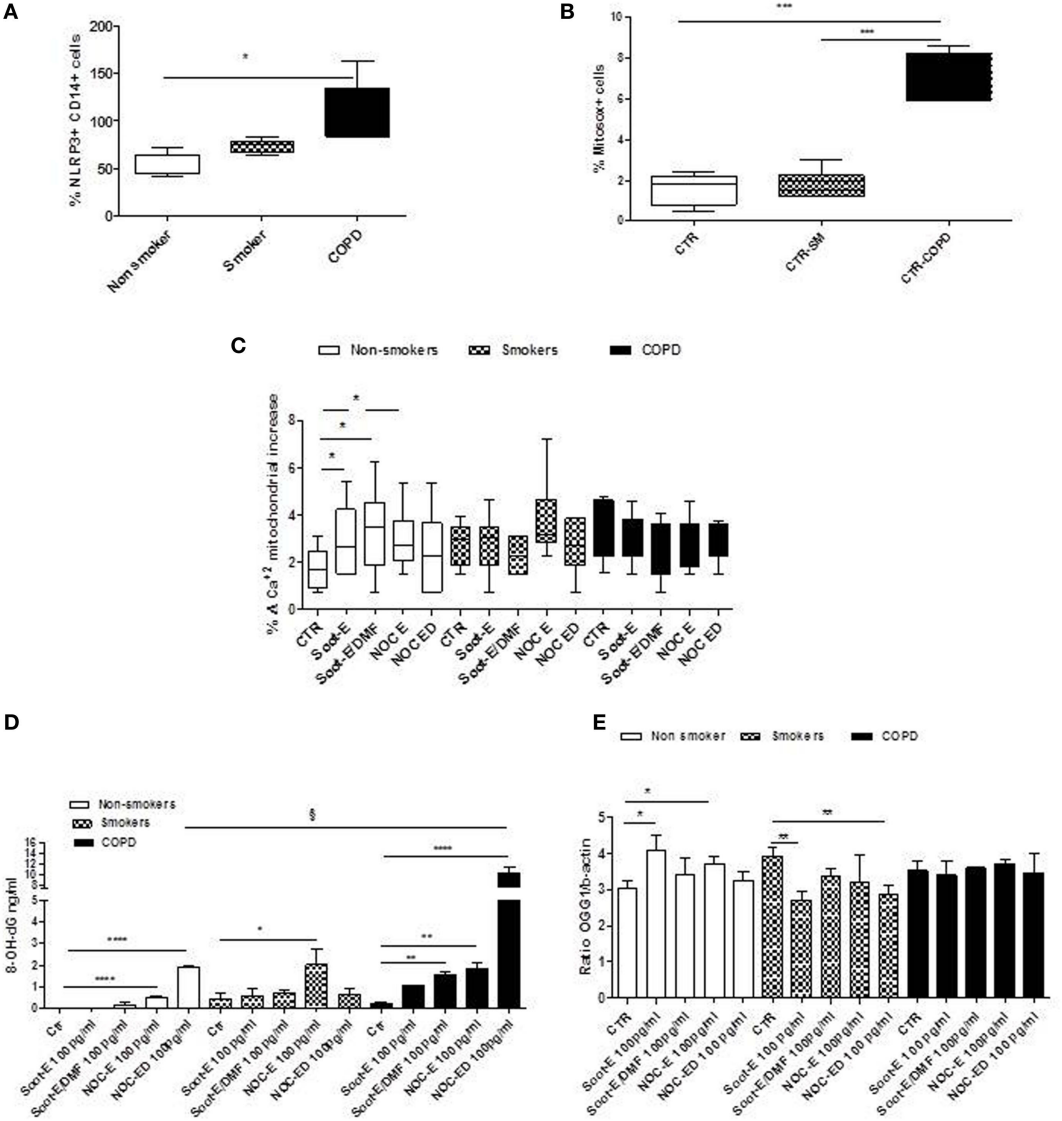
The administration of combustion-generated ultrafine particles (UFPs) induced mitochondria-dependent oxidative stress. Healthy non-smoker (white bars), smoker (dotted bars), and chronic obstructive pulmonary disease (black bars)-derived peripheral blood mononuclear cells (PBMCs) were treated with soot combustion-derived UFPs for 5 h. Levels of NOD-like receptor 3 (NLRP3) inflammasome **(A)** in CD14^+^ PBMCs and mitochondria-derived reactive oxygen species (mtROS), identified as Mitosox^+^ cells determined by means of flow cytometry **(B)**. **(C)** Levels of mitochondrial Ca^+2^ stores after the addition of Soot-E, Soot-E/DMF, nano organic carbon (NOC)-E, and NOC-ED at the concentration of 100 pg/ml for 1 h. **(D)** Levels of cytoplasmic 8-OH-dG after 3 h of treatment of PBMCs with Soot-E, Soot-E/DMF, NOC-E, and NOC-ED at the concentration of 100 pg/ml. **(E)** Levels of mRNA levels of OGG1 after 3 h of treatment of PBMCs with Soot-E, Soot-E/DMF, NOC-E, and NOC-ED at the concentration of 100 pg/ml, determined by means of RT-PCR. Control (CTR) represents untreated cells. Data are presented as median ± interquartile range **(A,B)** and means ± SEM **(C–E)** (*n* = 7). Statistically significant differences were determined by one-way analysis of variance followed by Bonferroni’s multiple comparison posttest. *, **, ^§^, ***, and **** represent *p* < 0.05, *p* < 0.01, *p* < 0.001, *p* < 0.0005, and *p* < 0.0001, respectively.

In our previous study, we demonstrated that the release of IL-1-like cytokines after UFP exposure was caspase-1- and NLRP3 inflammasome dependent in PBMCs from healthy smokers (11). To understand the molecular mechanism underlying the release of IL-18 and IL-33 from unstable COPD patients after organic UFP exposure, we carried on evaluating the role of NLRP3 and mitochondrial-dependent oxidative stress. We observed that the expression of NLRP3 in CD14^+^ PBMCs in basal conditions was significantly higher in COPD patients (Figure 3A, black bars) than non-smokers (Figure 3A, white bars) and smokers (Figure 3A, dotted bars). Similarly, mitochondrial-derived reactive oxygen species (mtROS) (identified as % Mitosox cells) were robustly higher in PBMCs from unstable/exacerbated COPD patients than non-smokers and smokers (Figure 3B). Starting from these observations and based on the concept that NLRP3 activation is strictly dependent on mtROS production (21), we measured mitochondria homeostasis under UFP treatment. The exposure to UFPs of PBMCs from non-smokers (Figure 3C, white bars) and smokers (Figure 3C, dotted bars) increased the release of calcium (Ca^+2^) from mitochondria only after an external stimulus (carbonyl cyanide p-trifluoromethoxy-phenylhydrazone, FCCP) during the measurement/detection, implying that UFP treatment did not alter mitochondrial calcium stores. By contrast, the measurement of Ca^+2^ stores in the mitochondria of unstable COPD-derived PBMCs were lower (Figure 3C, black bars) than those observed in non-smokers and smokers (Figure 3C, white and dotted bars) after Soot-E, Soot-E/DMF, NOC-E, and NOC-ED (100 pg/ml) treatment. The above data imply that the stimulation of PBMCs with these particles had already induced the release of Ca^+2^ from the mitochondria in unstable COPD-derived PBMCs. Because the release of Ca^+2^ from the mitochondria is strictly correlated to the release of mtROS (20) and protein/DNA damage, the levels of 8-hydroxy-deoxyguanosine (8-OH-dG), a well-known marker for DNA damage derived from oxidative stress (22), were measured. Very interestingly, the addition of NOC-E and NOC-ED significantly increased the release of 8-OH-dG in non-smoker (Figure 3D, white bars) and smokers (Figure 3D, dotted bars). However, 8-OH-dG release was more pronounced in PBMCs from unstable/exacerbated COPD patients after the addition of Soot-E/DMF, NOC-E, and NOC-ED (100 pg/ml) (Figure 3D, black bars).

To note, we did not observe a significant increase in mtROS from non-smokers after the addition of all particle samples (Soot-E, Soot-E/DMF, NOC-E, and NOC-ED, each 100 pg/ml) compared with smokers and COPD PBMCs (data not shown). Therefore, we went on to analyze the levels of a repairing enzyme, 8-oxoguanine glycosylase, OGG1, highly important to avoid DNA damage following oxidative stress (23). The administration of all UFPs (100 pg/ml) significantly increased the levels of mRNA in PBMCs obtained from non-smokers (Figure 3E, white bars). By contrast, PBMCs from smokers did not show any increase compared with the basal levels of mRNA for OGG1 after all UFP (100 pg/ml) exposure (Figure 3E, dotted bars), but rather, a significant decrease was observed. Interestingly, PBMCs from unstable COPD patients showed no increase of OGG1 mRNA levels after Soot-E, Soot-E/DMF, NOC-E, and NOC-ED (100 pg/ml) exposure (Figure 3E, black bars).

These data imply that treatment of PBMCs obtained from unstable/exacerbated COPD patients with Soot-E, Soot-E/DMF, NOC-E, and NOC-ED leads to mitochondrial dysfunction in that oxidative stress occurs. In this context, however, this effect is not countered by repairing enzymes, such as OGG1, establishing a pro-inflammatory/prooxidative cytoplasmic molecular pattern that leads to the release of IL-18 and IL-33 from PBMCs obtained from unstable/exacerbated COPD patients.

### The Release of IL-18 and IL-33 from Unstable COPD-Derived PBMCs Is Not NLRP3/Caspase-1-Dependent after Combustion-Generated UFP Exposure

Because the release of mtROS and the presence of 8-OH-dG are able to induce the activation of NLRP3 inflammasome and because we found that NLRP3 protein levels are higher in unstable COPD patients, we went on by analyzing the molecular mechanism underlying the release of IL-18 and IL-33 from unstable COPD-derived PBMCs after combustion-generated UFP exposure. Therefore, unstable COPD-derived PBMCs were treated with pharmacological inhibitors for caspase-1, ac-Y-Vad (Y-Vad, 1 µg/ml) (20), for NLRP3, glybenclamide (Gly, 1 µM) (11, 20) and for caspase-8, z-IETD-fmk (IE, 0.5 µg/ml) (24, 25) together with combustion-generated UFPs.

**Figure 4:**
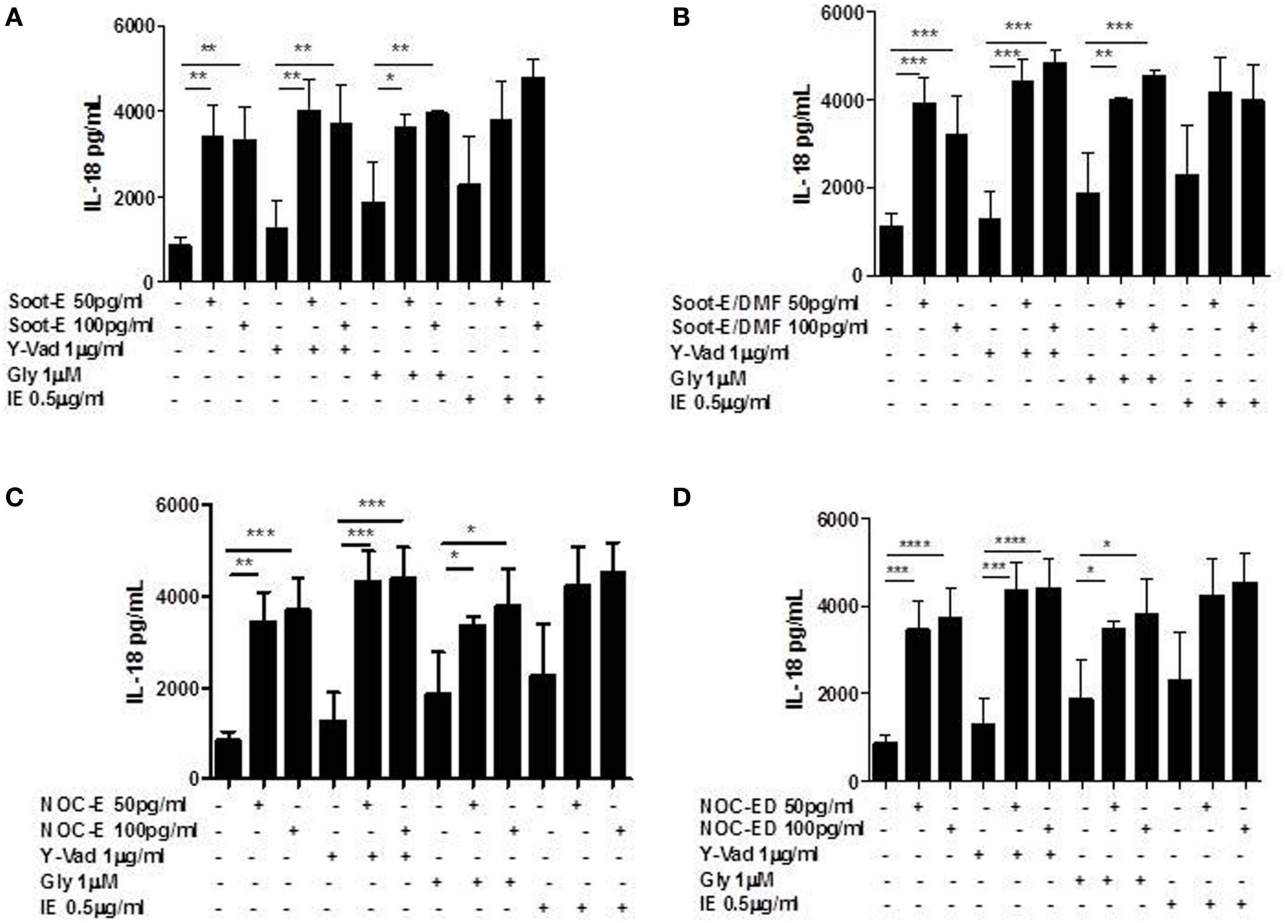
The release of IL-18 from chronic obstructive pulmonary disease (COPD) peripheral blood mononuclear cells (PBMCs) treated with combustion-generated ultrafine particles (UFPs) was not NOD-like receptor 3 (NLRP3)/caspase-1 and caspase-8-dependent. COPD (black bars)-derived PBMCs were treated with soot combustion-derived UFPs for 5 h in the presence of y-Vad, caspase-1 inhibitor (1 µg/ml), glybenclamide (Gly), NLRP3 inhibitor (1 µM), and IE (0.5 µg/ml) a caspase-8 inhibitor. The levels of IL-18 after **(A)** Soot-E, Soot-E/DMF **(B)**, nano organic carbon (NOC)-E **(C)**, and NOC-ED **(D)** treatment was not reduced in the presence of Y-Vad, Gly, and IE. Data are presented as the means ± SEM (*n* = 7). Statistically significant differences were determined by one-way analysis of variance followed by Bonferroni’s multiple comparison posttest. *, **, ***, and **** represent *p* < 0.05, *p* < 0.01, *p* < 0.0005, and *p* < 0.0001, respectively.

The pharmacological inhibition of caspase-1 did not alter the levels of IL-18 from unstable COPD-derived PBMCs after Soot-E (Figure 4A), Soot-E/DMF (Figure 4B), NOC-E (Figure 4C), or NOC-ED (Figure 4D) exposure. In support, the inhibition of NLRP3 by means of Gly did not affect the levels of IL-18 from unstable COPD-derived PBMCs after Soot-E (Figure 4A), Soot-E/DMF (Figure 4B), NOC-E (Figure 4C), or NOC-ED (Figure 4D) exposure. Moreover, the pharmacological inhibition of caspase-8, another enzyme involved in the non-canonical inflammasome-dependent pathway (26), did not alter IL-18 levels (Figures 4A–D).

**Figure 5:**
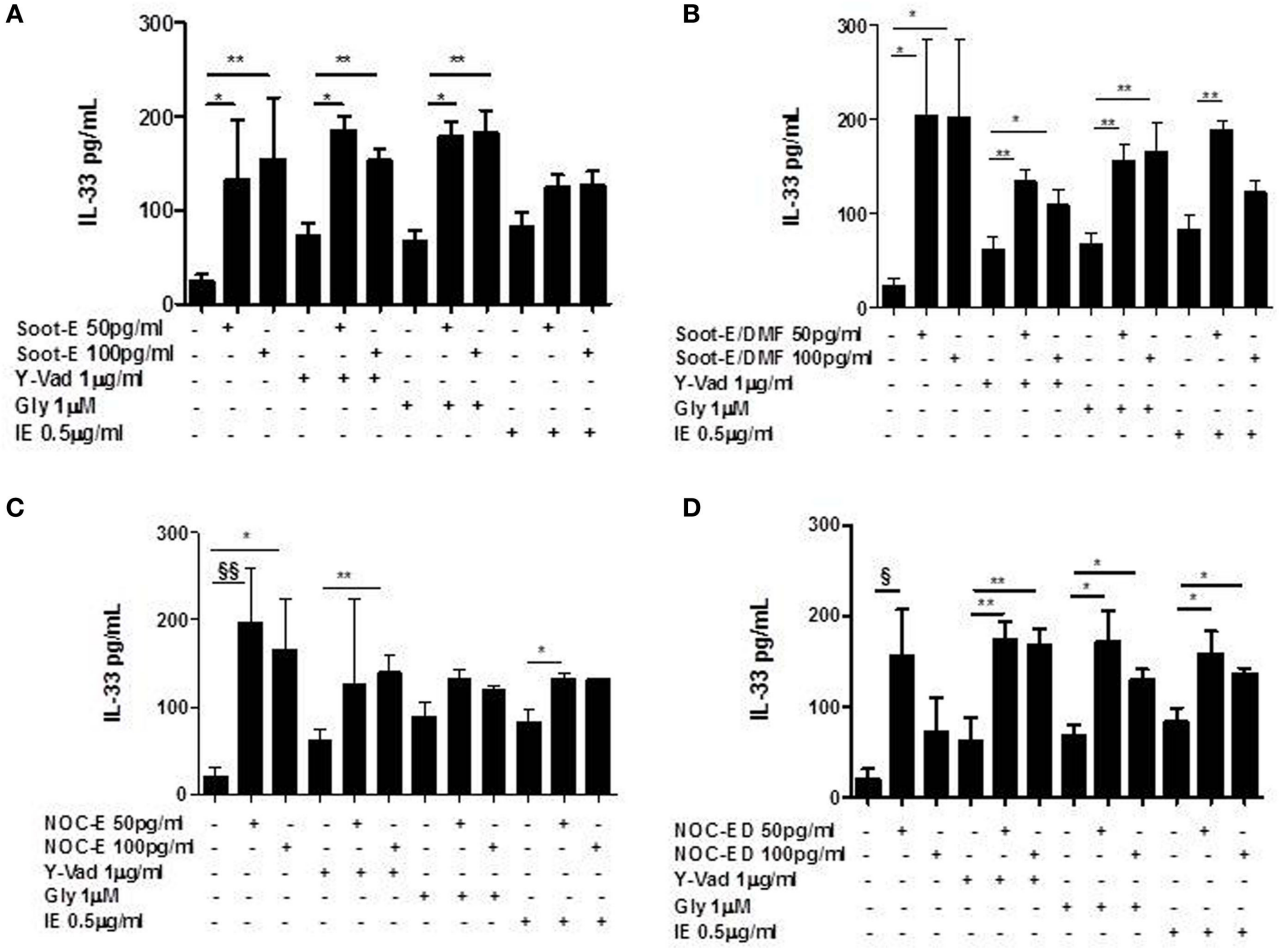
The release of IL-33 from chronic obstructive pulmonary disease (COPD) peripheral blood mononuclear cells (PBMCs) treated with combustion-generated ultrafine particles (UFPs) was not NOD-like receptor 3 (NLRP3)/caspase-1 and caspase-8-dependent. COPD (black bars)-derived PBMCs were treated with soot combustion-derived UFPs for 5 h in the presence of y-Vad, caspase-1 inhibitor (1 µg/ml), glybenclamide (Gly), NLRP3 inhibitor (1 µM). and IE (0.5 µg/ml) a caspase-8 inhibitor. The levels of IL-33 after **(A)** Soot-E, Soot-E/DMF **(B)**, nano organic carbon (NOC)-E **(C)**, and NOC-ED **(D)** treatment was not reduced in the presence of Y-Vad, Gly, and IE. Data are presented as the means ± SEM (*n* = 7). Statistically significant differences were determined by one-way analysis of variance followed by Bonferroni’s multiple comparison posttest. *, **, ^§^, and ^§§^ represent *p* < 0.05, *p* < 0.01, *p* < 0.005, and *p* < 0.001, respectively.

The same results were obtained for IL-33, where we observed that neither the inhibition of caspase-1 (Figures 5A–D) nor the inhibition of NLRP3 and caspase-8 (Figures 5A–D) altered the levels of the cytokine released from PBMCs obtained from unstable COPD patients exposed to combustion-generated UFPs.

### The Exposure of Unstable COPD-Derived PBMCs to Combustion-Generated UFPs Induced the Release of Caspase-4

Non-canonical inflammasome was also described as caspase-4-dependent in humans (caspase-11 in mice) (26). However, caspase-11 was defined as inducible, not constitutive, in mice (27). Therefore, we first analyzed the levels of mRNA for caspase-4 in PBMCs obtained from unstable COPD patients.

**Figure 6:**
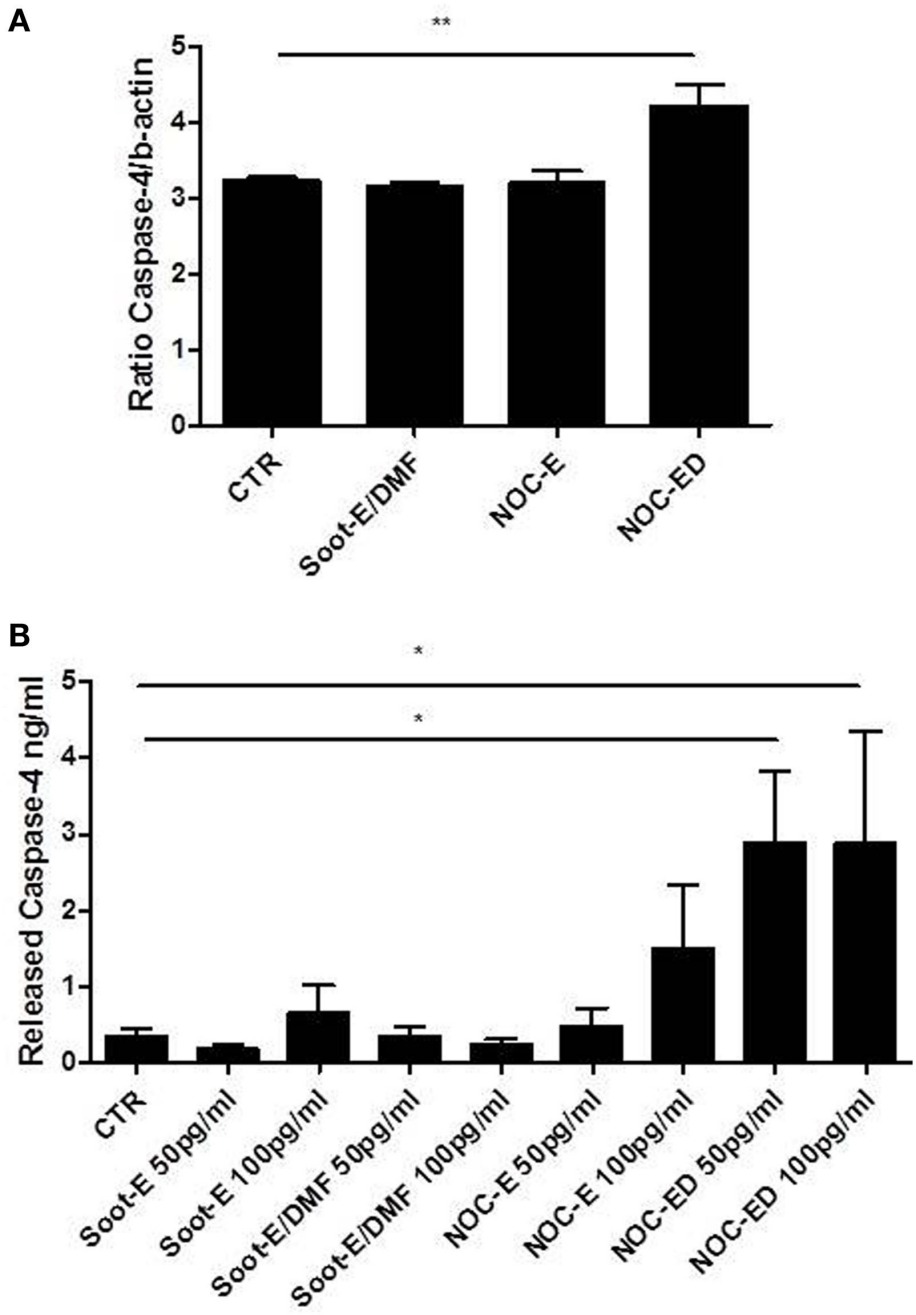
The stimulation of unstable chronic obstructive pulmonary disease (COPD)-derived peripheral blood mononuclear cells (PBMCs) with combustion-generated ultrafine particles (UFPs) induced caspase-4 release. COPD (black bars)-derived PBMCs were treated with soot combustion-derived UFPs for 5 h. The levels of caspase-4 mRNA were determined **(A)** after Soot-E/DMF, nano organic carbon (NOC)-E, and NOC-ED treatment. The release of caspase-4 **(B)** was evaluated by means of a patented ImmunePharma’s ELISA kit. Control (CTR) represents untreated cells. Data are presented as the means ± SEM **(A)** and **(B)** (*n* = 7). Statistically significant differences were determined by one-way analysis of variance followed by Bonferroni’s multiple comparison posttest. * and ** represent *p* < 0.05 and *p* < 0.01, respectively.

We found that caspase-4 mRNA levels were detectable in CTR cells and, interestingly, the treatment of cells with NOC-ED significantly increased caspase-4 mRNA levels (Figure 6A). To evaluate the potential involvement of caspase-4 in UFP effects, we used a patented ELISA kit (as described in Section “Materials and Methods”). Very interestingly, the addition of NOC-ED significantly increased the release of caspase-4 from PBMCs obtained by unstable COPD patients (Figure 6B). However, we were not able to reach a statistical difference when cells were treated with the other three UPFs samples, Soot-E or Soot-E/DMF or NOC-E (Figure 6B). Nevertheless, a potential increase was observed: 0.61 ± 0.32 ng/ml for Soot-E (100 pg/ml) vs 0.35 ± 0.09 ng/ml for CTR; 1.36 ± 0.74 ng/ml for NOC-E (100 pg/ml) vs 0.35 ± 0.09 ng/ml for CTR. Similar data were observed when COPD-derived PBMCs were treated with coronene (10 pg/ml; 1.74 ± 0.7 ng/ml).

Taken together, these data imply the extracellular release of caspase-4 and its involvement in IL-18 and IL-33 increase when PBMCs obtained from unstable COPD patients are treated with UFPs.

## Discussion

In this study, we found that combustion-generated UFPs induced mitochondrial-derived oxidative stress, which is not countered by the enzyme OGG1, deputed to repairing oxidative stress damage, leading to the release of IL-18 and IL-33 from PBMCs obtained from unstable/exacerbated COPD patients. Importantly, the release of IL-18 and IL-33 was not dependent on the activation of the canonical, caspase-1-dependent, and non-canonical, caspase-8-dependent, inflammasome pathway but rather on the release of the caspase-4, which activity still remains to be defined after UFP exposure. By contrast, PBMCs obtained from stable COPD patients were not affected by the exposure to this particulate matter in terms of IL-18 and IL-33 release.

Literature data report that high levels of IL-1β and IL-18 are usually found in the lungs of patients with COPD after cigarette smoke exposure, implying the involvement of the inflammasome in this pathology (1, 7, 13). In support, the adaptor protein ASC was found at high levels and in a speck-like form in the sputum of COPD patients (12). Experimental COPD animal models showed that the genetic absence of both caspase-1, the enzyme involved in the canonical inflammasome complex, and NLRP3 reduce COPD-like features in mice exposed to cigarette smoking (5, 9, 10, 12), further confirmed by neutralization of IL-1β. However, published data on human samples from COPD patients are controversial in that no correlation between NLRP3, caspase-1, and IL-1β responses was observed when comparing stable COPD patients with smokers (1). These data imply that the inflammasome is not triggered in these clinical conditions, probably explaining the discrepancy in literature and the unsuccessful randomized clinical trials performed on stable COPD patients subjected to canakinumab (monoclonal antibody against IL-1β) or an anti-P2X7 antagonist (1). To note, most of the human studies were performed on stable COPD-derived samples. In sharp contrast, our study was focused on PBMCs from unstable/exacerbated COPD patients, and we found that the sole IL-18 and IL-33, but not IL-1α and IL-1β (undetectable), were released after the exposure to combustion-generated UFPs. Instead, stable COPD-derived PBMCs were not responsive to combustion-generated UFPs in terms of IL-18 and IL-33 release (data not shown). This is, to the best of our knowledge, the first study showing that unstable COPD-derived PBMCs were able to release IL-1-like cytokines in a non-canonical inflammasome-dependent manner after combustion-generated UFPs exposure. In support, as proposed by Di Stefano et al., NLRP3, Caspase-1 and IL-1β responses in exacerbated/unstable COPD patients would be relevant (15). We found that although, the higher levels of NLRP3 in the PBMCs of these patients, it was not involved in the release of IL-18 and IL-33 after combustion-generated UFP exposure. However, in our previous study, we found that the stimulation of smoker-derived PBMCs with pyrolytic combustion-derived UFPs released higher IL-1-like cytokines in a NLRP3-dependent manner (11). Because all the PBMCs we used were obtained from smoking or former smoking COPD patients, we expected that NLRP3 inflammasome was most likely leading to the chronic inflammatory response typical of COPD after combustion-generated UFP exposure. Instead, we found that smokers were less responsive to combustion-generated UFPs than COPD patients, implying that the nature of air pollutants is of great relevance. Moreover, it is likely that although the higher levels, NLRP3 may be impaired in PBMCs of unstable COPD patients (11, 15), explaining the discrepancies in the literature. Indeed, the pharmacological inhibition of NLRP3 by means of Gly did not alter IL-18 and IL-33 levels. Similarly, we did not observe any inhibition of these cytokines when we inhibited caspase-1 and caspase-8, which underlie NLRP3 activation in a canonical and non-canonical manner, respectively (26). It is to note that although the inhibition of caspase-8 did not alter IL-18 production after UFPs addition (Figures 4A–D), we were not able to reach a statistical difference between the basal levels when the sole caspase-8 inhibitor was added, versus the levels of the cytokine when the inhibitor and UFPs were added (Figure 4D). This effect was not observed for IL-33 (Figure 5).

By contrast, we found that caspase-4 release occurred after the exposure of PBMCs obtained from unstable COPD patients to combustion-generated UFPs, implying its involvement in IL-18 and IL-33 release. It is to point out, though, that we cannot assume by these data that caspase-4 was directly involved in IL-18 and IL-33 release due to the absence of a commercially available specific inhibitor of the sole caspase-4. Moreover, we have to highlight that stable COPD-derived PBMCs treated with combustion-generated UFPs did not induce caspase-4 release. Similarly we did not detect any difference compared with the basal conditions of release of IL-18 and IL-33 from PBMCs obtained from stable COPD patients treated with UFPs. These data further underlie the relevance of inflammasome-dependent mechanisms in stable vs unstable COPD patients.

Another important issue is the role of the oxidative stress in PBMCs from unstable COPD patients. Oxidative stress is a critical feature and key mechanism in many molecular processes during the onset of COPD (1). Mitochondrial dysfunction leads to excessive production of mtROS resulting in harmful effects, such as damage to lipids, proteins, and DNA. In this study, we found that unstable COPD-derived PBMCs exposed to combustion-generated UFPs had higher mitochondrial impairment in that higher levels of oxidized nucleic acids (i.e., 8-OH-dG, widely used as a marker), were found compared with non-smoker PBMCs. 8-OH-dG levels in unstable COPD-derived PBMCs were even higher than those observed in smoker-derived PBMCs after UFP exposure. This effect was strictly dependent on the expression of OGG1, an enzyme involved in the repair of DNA damage following mitochondrial dysfunction (23). OGG1 was not higher in the PBMCs of unstable COPD patients and even more important, it was not increased after combustion-generated UFP exposure, focusing on the clinical outcome/lifestyle of these patients when exposed to both indoor and outdoor pollution. Moreover, because of the strict correlation between NLRP3 activation and mtROS/oxidized DNA derivatives, it was obvious to believe that NLRP3 was involved. Instead, the pharmacological inhibition of NLRP3 did not show any correlation with IL-18 and IL-33 release after combustion-generated UFP exposure. In this context, though, we have to highlight that as reported by Shimada et al. (21), 8-OH-dG can bind to NLRP3 avoiding the activation of the inflammasome. Therefore, it is likely that NLRP3 is not involved in UFP-induced IL-18 and IL-33 release because of its inhibition by 8-OH-dG. More importantly, we found that caspase-4 is released into cell-free supernatant. The biological role of the released caspase-4 from unstable COPD-derived PBMCs is still under-investigated, and it still remains to investigate how caspase-4 is involved in IL-18 and IL-33 levels from PBMCs of unstable COPD patients.

In conclusion, our study highlights a novel molecular mechanism by which combustion-generated nanoparticles induce the release of the pro-inflammatory IL-18 and IL-33 in a non-canonical caspase-4-dependent inflammasome pathway from PBMCs of unstable/exacerbated COPD patients. Our study opens new perspectives in the field of the inflammasome in COPD during the exacerbation of clinical conditions, when the active form of caspase-4 is more relevant after cigarette smoke and air pollution exposure, paving the way for alternative pathways to be pharmacologically triggered in COPD pathogenesis.

## Ethics Statement

Human samples: blood from COPD and non-COPD subjects were collected at the Hospital “Monaldi-Azienda Ospedaliera (AORN)-Ospedale dei Colli” in Naples, Italy, after signed informed consent. The experimental protocol was performed in accordance with the guidelines and regulations provided and accepted by the Ethical Committee of the “Monaldi-AORN-Ospedale dei Colli” Hospital (approval number 1254/2014).

## Author Contributions

GF, CC, MT, MS, MC, APinto, MP, AD, RA, PM, and RS designed the experimental protocol. GF, CC, MT, APopolo, and MP performed the experiments. MC and MS prepared the soot particle samples. AD, RA, APinto, and RS interpreted the data and wrote the manuscript. All the authors read and approved the final manuscript.

## Conflict of Interest Statement

The authors declare that the research was conducted in the absence of any commercial or financial relationships that could be construed as a potential conflict of interest.

## Supplementary Material

Figure S1 The stimulation of healthy non-smoker [white bars **(A)**], smoker [dotted bars **(B)**], and chronic obstructive pulmonary disease [black bars **(C)**] with coronene (10 pg/ml) or pyrene (10 pg/ml) induced the release of IL-18. Control (CTR) represents untreated cells. Data are presented as the means ± SEM (*n* = 7). Statistically significant differences were determined by one-way analysis of variance followed by Bonferroni’s multiple comparison posttest. * represents *p* < 0.05.
