# Supplementary figures and images for: Chronic Obstructive Pulmonary Disease-Derived Circulating Cells Release IL-18 and IL-33 under Ultrafine Particulate Matter Exposure in a Caspase-1/8-Independent Manner
Source: Front Immunol. 2017 Oct 26;8:1415. doi: 10.3389/fimmu.2017.01415 (PMC5662642; doi:10.3389/fimmu.2017.01415)

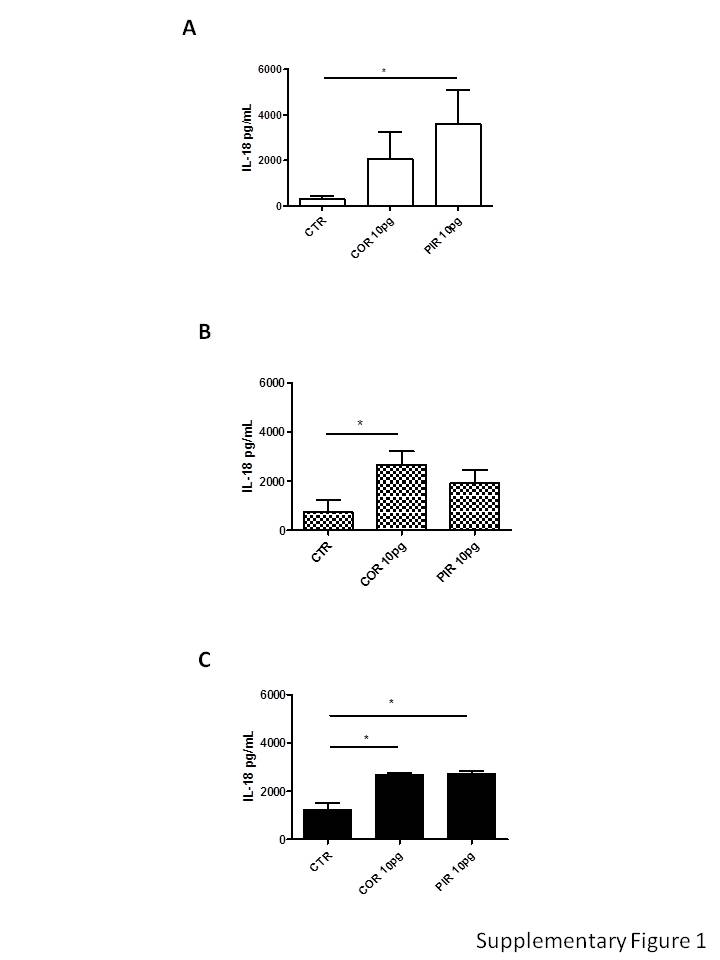

Supplement: Figure S1 — The stimulation of healthy non-smoker [white bars (A)], smoker [dotted bars (B)], and chronic obstructive pulmonary disease [black bars (C)] with coronene (10 pg/ml) or pyrene (10 pg/ml) induced the release of IL-18. Control (CTR) represents untreated cells. Data are presented as the means ± SEM (n = 7). Statistically significant differences were determined by one-way analysis of variance followed by Bonferroni’s multiple comparison posttest. * represents p < 0.05. [file image_1.jpeg]
